# Supplementary material for: Targeting Aging and Longevity with Exogenous Nucleotides (TALENTs): Rationale, Design, and Baseline Characteristics from a Randomized Controlled Trial in Older Adults
Source: Nutrients. 2024 Apr 29;16(9):1343. doi: 10.3390/nu16091343 (PMC11085046; doi:10.3390/nu16091343)
Supplement: Supplementary file 1 [file nutrients-16-01343-s001.zip › nutrients-2958372-supplementary.pdf]

SUPPLEMENTAL MATERIAL

Appendix A.The biospecimen collection of the TALENTs trial.

Appendix B. The informed consent of the TALENTs trial.

Appendix C. The Comprehensive Geriatric Assessment Questionnaire of the TALENTs trial.

Appendix A.The biospecimen collection of the TALENTs trial.

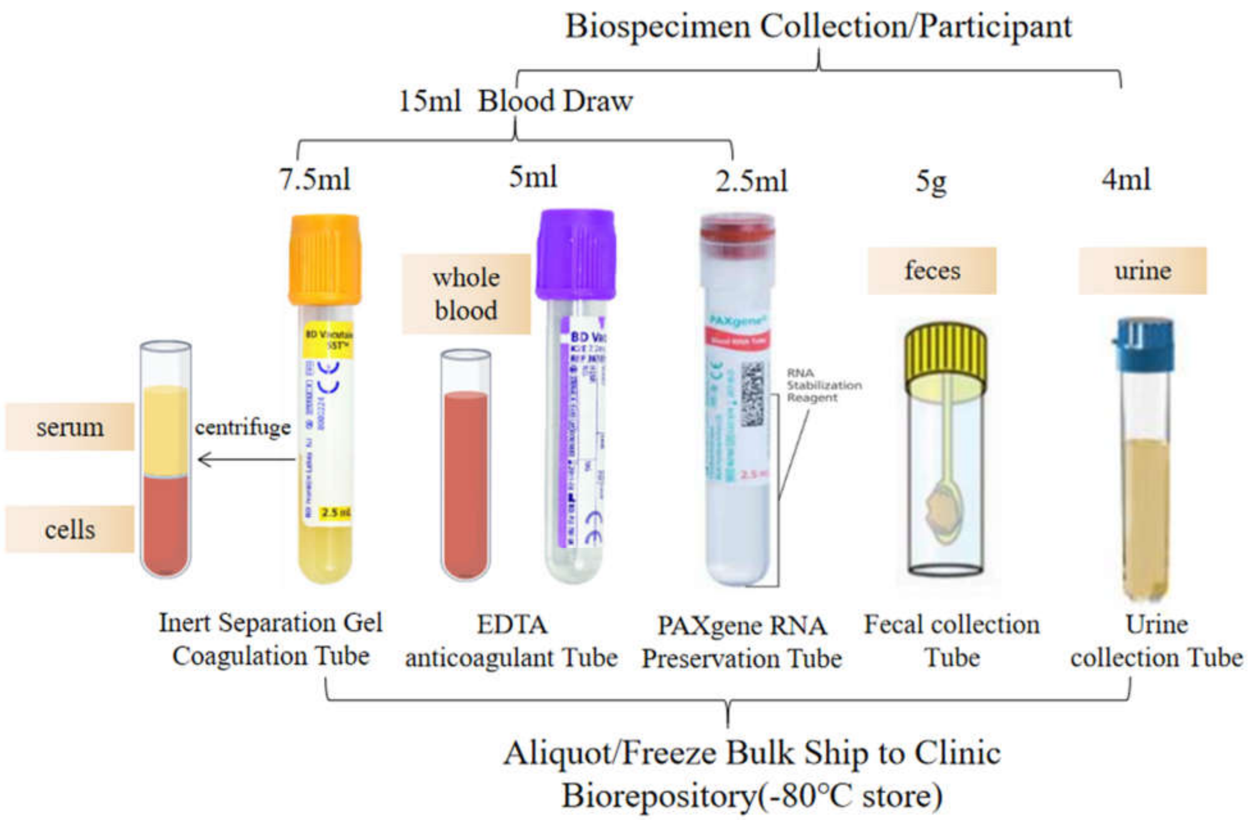

Appendix B. The informed consent of the TALENTs trial.

The TALENTs trial (Targeting Aging and Longevity with Exogenous Nucleotides): Informed consent

Dear resident：

We are going to carry out a clinical trial study on the Anti-aging effect of Exogenous nucleotides, and you meet the enrollment conditions of this study, so we would like to invite you to participate in this study. This informed consent will introduce to you the purpose, steps, benefits, risks, inconvenience or discomfort that may be caused to you, etc. Please read it carefully before making a decision on whether to participate in the study. When the researcher explains and discusses the informed consent to you, you can always ask questions and let him/her explain to you what you don't understand. You can discuss this with family, friends, and your doctor before making a decision. The study was led by Meihong Xu, an associate researcher at Peking University's School of Public Health. This study was commissioned and funded by Hainan Shuangdi Zhenao Life Science Research Center Co., LTD.

1. Why did this study take place?

With the increase of age, many tissues and organs of the body are declining, which brings great challenges to the health of the elderly. Finding effective food active ingredients to anti-aging, prevent the occurrence of age-related diseases, and improve the health of the elderly has become a major topic in the field of life science research. Exogenous nucleotides have high nutritional value and health care function. Human experimental studies have found that it has the function of regulating immunity and promoting growth and development. At the same time, the results of previous animal experiments showed that nucleotide supplementation can regulate intestinal flora, antioxidant, relieve physical fatigue, assist in lowering blood lipids, protect alcoholic liver injury, assist in improving memory and other health effects, and has the potential to anti-aging and improve the health status of the elderly. The purpose of this study was to explore the anti-aging effect of exogenous nucleotides in human body and its mechanism.

2. Who will be invited to participate in the study?

You are invited to participate in this study because you are: between 60 and 70 years of age; In good health with no serious physical or mental illness; Never taken a

nucleotide supplement (including health food/health food); Be able to fully comply with the requirements of this study and complete the study enrollment conditions for the relevant assessment.

3. How many people will participate in the study?

The study plans to enroll 120 participants. Among them, 60 were placebo control group and 60 were nucleotide test group.

4. How was the study conducted?

This study intends to use a randomized, controlled, double-blind trial method to conduct a 4-month human intervention trial. You will be randomly assigned to one of two groups, the placebo control group or the nucleotide intervention group. Take the test capsules for this group. Randomization means "you will be randomly assigned (like flipping a coin) to either of the two groups (taking a plain placebo or nucleotide)." You have a 50-50 chance of using the study subject Placebo is a common starch that is safe for humans but has no health benefits. This is a double-blind study and you will not be able to know your group throughout the study. At the end of the trial we will unblind. After unblinding, understanding that you are assigned to the placebo control group, you will be compensated with the same amount of nucleotide capsules as the nucleotide group during the trial.

At the beginning of the experiment, 2 months and 4 months after the experiment, we will carry out the following specific work, and we need your cooperation:

1) Complete the questionnaire survey, including the basic information, diet, lifestyle and behavior survey;

2) Physical examination: unified arrangement of physical examination, physical assessment and B ultrasound/color ultrasound, blood and stool tests;

3) In this study, 27mL blood (about 6 blood vessels depending on the volume of blood vessels) should be drawn, 12mL of which is used for the detection of blood routine, blood biochemistry, liver function, kidney function, immune function, glycated hemoglobin, insulin, C-reactive protein, cancer markers and other indicators; 15mL blood samples were used to detect age-related indicators and sequence age-related genes, including oxidative stress, mitochondrial function and other indicators; FOXO and APOE4 genotypes; Transcriptional expression of KAT7, IGR-1 and glutaminase genes. During the whole test, blood was drawn 3 times in total. The blood test will be

carried out in a qualified unit (such as a hospital), and the relevant operation will be completed by an experienced nurse and kept as a sample.

4) In this study, in addition to routine stool inspection, 2g of stool samples (about 1-2 spoons) should be collected for microflora sequencing.

We will get back to you with the test results. If necessary, we can make targeted recommendations to you based on your information and test results. You will undergo a medical examination throughout the study and all original laboratory results will be kept by us.

5. How does participating in the study affect the subjects' daily life?

When you decide whether to participate in this study, please carefully consider the impact of the tests and follow-up listed above on your daily work, family life, etc. Consider the time and transportation for each return visit. If you have any questions about the tests and procedures involved in the trial, please contact us. Your lifestyle and medications should remain as unchanged as possible during the trial. Consult your study physician before taking any new prescribed medications. For your safety and to ensure the validity of the study results, you may not participate in any other clinical study during the study period.

6. What are the risks and adverse reactions of participants in this study?

The risks that may be associated with your participation in this study are as follows. You should discuss these risks with your family, or if you wish, with the doctor who cares for you on a regular basis.

Nucleotide risk: As a food raw material approved by the State Food and Drug Administration, nucleotide has been widely used in infant formula milk powder/food, health food/special medical use formula food, high safety. However, there may still be risks, discomfort, drug interactions or adverse reactions that are currently unknown due to individual differences.

Risks of drawing blood: The risks of drawing blood from the arm include temporary discomfort and/or bruising. Although highly unlikely, infection, excessive bleeding, clotting, or fainting may occur.

Information security risks: We will do our best to protect the information you provide from disclosure, however, we cannot guarantee the absolute security of the information. Blood and stool samples are sent for immediate testing, not retained, and no genetics-related studies are planned. Biological samples and related data were used

for this study only. The relevant survey data will be stored in the research group until five years after the completion of the project.

Dr. Guo Xianyu, chief physician of the research project team, will serve as a consulting expert and provide you with necessary medical consultation in case of adverse reactions related to the study; Severe cases should be treated in the nearest medical hospital.

7. What are the possible benefits of participants in this study?

There are no direct benefits to this study. By exploring the anti-aging effect of exogenous nucleotides and its mechanism, this study may provide new ideas for prolonging healthy life.

8. Is it necessary to participate in and complete the study?

Your participation in this study is entirely voluntary. If you do not wish, you may refuse to participate without any negative impact on your current or future health care. Even after you agree to participate, you can change your mind at any time and tell the investigator to withdraw from the study. We will inform you of any information that may affect your decision to continue to participate in the study. The sponsor or regulatory authority may also terminate the study during the study period. In the event of an early termination of the study, you will be notified and your study physician will advise you on the next treatment plan based on your health condition. In principle, after you opt out, the researcher will keep your relevant information closely until the final destruction, during which the information will not continue to be used or disclosed. However, in the following rare cases, researchers will continue to use or disclose information about you even after you have withdrawn from the study or the study has ended. These include: Removal of your information will affect the scientific nature of the research results or the evaluation of data security; Provide limited information for research, teaching or other activities (this information will not include your name, ID number, or other personal information that identifies you); When schools and government regulators need to monitor research, they will ask to see all research information, including information about your involvement in the research at the time.

9. Who will bear the cost of participating in the study?

The entire cost of physical examination, comfort capsules/nucleotide intervention capsules for this study is the responsibility of the project side, and you do not have to pay any cost.

10. Are participants paid to participate in the study?

Participants in this study are compensated with 500 RMB for transportation and other expenses. The compensation will be paid out in cash as a lump sum after the final medical examination (4 months after the trial starts).

11. Will the subject's personal information be kept confidential?

If you decide to participate in the study, your participation in the study and your personal data in the study will be kept confidential. Your blood/urine specimen will be identified by the study number and not by your name. Information that can identify you will not be disclosed to members outside the research group unless you have given your permission. All research members and research sponsors are requested to keep your identity confidential. Your files will be kept in a locked filing cabinet and will only be accessible to researchers. In order to ensure that the research is carried out in accordance with the regulations, the members of the government administration or ethics committee can access your personal data in the research unit as required. No personal information about you will be disclosed when the results of this research are published. If there is any information that may affect your continued participation in the trial, you will be notified in a timely manner. You have the right to withdraw your consent/authorization; The right to refuse to sign the consent form/power of attorney.

12. Who should I contact if I have a problem or difficulty?

If you have any questions related to this study, please contact Meihong Xu, Associate Researcher, School of Public Health, Peking University, during working hours (office number: 010-82805243), after work hours, weekends and holidays (mobile number: 15101035121,18810515491). If you have any questions concerning the rights and interests of subjects, you may contact the Biomedical Ethics Committee of Peking University at 010-82805751 or llwyh@bjmu.edu.cn.

Investigator's statement：

"I have informed the subject of the research background, purpose, steps, risks and benefits of the Human Trial Study of Exogenous Nucleotide Anti-Aging Effects, and given him/her sufficient time to read the informed consent, discuss it with others, and answer his/her questions about the study; I have told the subject to contact Associate Researcher Xu Meihong at any time when he has problems related to the research, and to contact the Biomedical Ethics Committee of Peking University at any time when he has problems related to his own rights/interests, and provided accurate

contact information; I have informed the subject that he/she may withdraw from the study; I have informed the subject that he/she will be provided with a copy of this informed consent with my/her signatures."

Informed consent obtained by the investigator signature_________ Date___________

Subject statement:

"I have been informed of the background, purpose, procedure, risks and benefits of the study" Human Trial Study of Exogenous nucleotide Anti-Aging Effects. I had plenty of time and opportunity to ask questions, and I was satisfied with the answers. I was also told who to contact when I had questions, difficulties, concerns, suggestions for research, or wanted further information or help with research. I have read this informed consent form and agree to participate in this study. I know that I can withdraw from the study at any time during the study without any reason. I was told that I would get a copy of this informed consent form with my signature and that of the researcher."

Subject signature_____________________ Date____________________

Appendix C. The Comprehensive Geriatric Assessment Questionnaire of the TALENTs trial.

Comprehensive Geriatric Assessment Questionnaire (CGAQ) of the TALENTs trial

Dear resident:

The purpose of this project is to identify the anti-aging effects of exogenous nucleotides through randomized controlled trials and longitudinal monitoring at different time points. Your specific situation meets the eligibility criteria for this study, so we would like to invite you to participate in the study.

Questionnaire is a means of research in this project. In order to better present the research results, we invite you to help us complete the following questionnaire. Please answer the following questions independently and truthfully under questioning by the

investigator. There are no right or wrong answers, and each of your answers is very important to us. We are very eager to get your support and assistance.

Guarantee of confidentiality of respondents' personal information: We will keep all personal information collected in this survey strictly confidential. Your name and address records will only be used for future follow-up contact. The survey data file entered into the computer will not include the names and addresses of any individuals. Therefore, it is also impossible for anyone to identify any respondent from the computer survey data file. All questionnaires will be stored in the data warehouse.

TABLE OF CONTENTS

Personal Information........10

A: General information questionnaire........10

- A1 Basic Information........10
- A2 Nutritional Status........12
- A3 Medication Information........12

B: Health information questionnaire........13

- B1 Health Screening Questionnaire........13
- B2 Physical Activity Scale for the Elderly (PASE)........15
- B3 Fatigue Scale-14........18

C: Survey on sleep, emotions, and cognitive status........19

- C1 Pittsburgh Sleep Quality Index (PSQI)........19

D: Dietary questionnaire........24

- D1 Nutrition literacy questionnaire for the elderly........24
- D2 Food Frequency Questionnaire (FFQ)........27

Personal Information

Name: ___________________

Number (ID):______________

Date of birth: ______Year______Month _____

Date Age:______

Gender: ☐ Male ☐ Female

Skin status: ______________

Eyesight (including wearing glasses): ___________

Hearing:_________________

Residential address:________Provincial_____city (county)_____District (township)______

street (village)________Number

Contact Phone: __________________

A: General information questionnaire

A1 Basic Information

A1-1 Your nationality ( )

1. Han 2. Yi 3. Hui 4. Tibetan 5. Miao 6. Tujia 7. Mongolian 8. Qiang 9. Others (please specify:_)

A1-2 Your place of origin ( )

A1-3 Your level of education ( )

1.Did not go to school 2. Did not finish primary school 3. Primary school 4.unior high school 5. High school 6. Secondary technical school, vocational school 7. University or college 8. Master degree or above

A1-4 How many people have you lived with **in the last year**? ( )

1.Living alone 2. Living with your spouse 3. Living with your spouse and children 4. Other (please explain: __________) (Explanation: including: nursing homes, nannies, etc.)

A1-5 Your marital status ( )

1.Unmarried 2. At marriage 3. Divorced or separated 4. Widowed

A1-6 Your occupation is ( )

1. All kinds of professional and technical personnel 2. Heads of state organs, party and mass organizations, enterprises and institutions 3. Clerical and related personnel 4. Commercial staff

5. Service staff 6. Agricultural, forestry, animal husbandry and fishery workers 7. Production work, transportation work and some manual workers 8. Others (please specify:_______________)

A1-7 The average monthly income of this family (after tax)？ ( )

1. Below 2000 2. 2000-3500 3. 3501-5000 4. 5001-6500 5. 6501-10000 6. 10,000 yuan and above

A1-8 Do you smoke？ ( )

1. Never; (select this option, direct answer **A1-9**) 2. Had quit smoking (select this option, and continue to answer)____years, daily___root, quit___years; (Tip: If you cannot answer the number of roots, you can ask for the number of packets, i.e., 1 packet = 20 roots) 3. Smoking (Select this option and continue to answer) Smoking ______ years, daily ______ root (Hint: if you cannot answer the number of years, you can ask "Since when did you start smoking?" If the answer is that the number of cigarettes smoked per day is not fixed, or the number of smokers has decreased, you can add "the average number of cigarettes smoked per day in the past five years")

A1-9 Do you drink alcohol ？ ( )

1.Never; (Select this item and answer **A1-10** directly) 2. Have stopped drinking (select this item and continue to answer) Alcohol ______ twice a day, ______ years; (Hint: If the answer is to drink different kinds of alcohol, you can add "the number of drinks of each type" or "the number of drinks of the most frequently consumed type"; If you can't answer the number of drinks, you can ask "how many drinks", and you can ask the props of the cup) 3. (Hint: If you can't answer the number of years you have smoked, you can ask "Since when?")

A1-10 How many times have you eaten out **in the past month** (note: take-out, take-away) _____

A1-11 Are you taking health care products? ( )

1.No (Select this item and answer **A1-12** directly) 2. Yes (select this item and continue to answer) have been taking health care products for ______ years; The average annual cost is about _____ yuan. (Note: You can write the range of costs)

A1-12 Currently, you are enrolled in medical insurance situation is ( )

1.Basic medical insurance for urban employees 2. Medical insurance for urban residents 3. New rural cooperative Medical care 4. Free medical care 5. Commercial medical insurance

A1-13 Medical expenses **in the past 1 year**, the cost of physical examination is about__ RMB;The cost of medicine _yuan; (only for daily medicine) hospital expenses ______yuan; When was the last time you were hospitalized? Because_________________, the last hospitalization cost __________RMB

A2 Nutritional Status

A2-1 How much weight have you lost **in the last 3 months**? ( )

1. Weight loss greater than 3kg 2. Weight loss 1-3kg 3. No weight loss 4. I wonder

A2-2 Whether your food intake has changed **in the past 3 months**? ( )

1. The amount of food eaten has been severely reduced (reduced by 2/3, that is, by more than half). 2. Moderate reduction in food intake (by half) 3. Mild reduction in food intake (by 1/3) 4. No change in food intake 5. Serious increase in food intake (increased by 2/3, nearly twice the original) 6. Moderate increase in food intake (increase by half) 7. Mild increase in food intake (increase by 1/3)

A2-3 Do you cough or have difficulty swallowing? ( )

1. Yes 2. No

A3 Medication Information

For the medicines and health care products you have been taking **in the last 3 months**, please fill in the following information (see attached form) according to the packaging and instructions of your medicines or health care products.

Medicines/Health Care Products Usage Registration Form

Whether or not you have had any pre-existing medical conditions prior to the study? Yes☐ No☐, if yes, please fill in the name of the disease in the form below.

Number	Disease	Generic name for medicines/ health products	Medicines/ Health products manufacturers	Main components	Dosage per day	Start Date of Medicines/ health products (y/m/d)	End Date of Medicines/ health products (y/m/d)	Remarks
1								
2								
3								

4								
5								

Disease hints: heart disease, myocardial infarction stroke (stroke), kidney disease, malignancy, liver disease, hypertension, hypercholesterolemia, dyslipidemia (hyperlipidemia or hypolipidemia), autoimmune diseases, emphysema, chronic bronchitis, tuberculosis, asthma, peptic ulcers, gallstones or associated with cholecystitis, arthritis or rheumatism, neurasthenia, fractures, diabetes, chronic kidney disease, emotions, and mental problems, memory-related diseases (e.g., Alzheimer's, brain atrophy, Parkinson's disease)

B: Health information questionnaire

B1 Health Screening Questionnaire

Please answer according to your usual situation and choose the answer that best meets the actual situation.

B1-1 In general, how do you feel about your health? (   )

1. Excellent 2. Very good 3. Good 4. Average 5. Poor

B1-2 Does your current health condition limit your ability to engage in these activities? If so, to what extent?

B1-2-1 Moderate intensity activity (Like moving tables, vacuuming floors, or practicing tai chi) (   )

1. very restrictive 2. a little restrictive 3. no limits

B1-2-2 Climb the stairs (   )

1. very restrictive 2. a little restrictive 3. no limits

B1-3 Have you had any of the following problems **in the past week** because of your health?

B1-3-1 Are you actually getting less done than you want? (   )

1. Yes 2. No

B1-3-2 Is the type of work or other activity restricted? (   )

1. Yes 2. No

B1-4 **In the past week**, during work or other daily activities, have you experienced any of the following problems due to emotional reasons (such as feeling depressed or anxious)?

B1-4-1 Are you actually getting less done than you want? (   )

1. Yes 2. No

B1-4-2 Less careful than usual at work or other activities? (   )

1. Yes 2. No

B1-5 **In the past week**, how much does your physical pain affect your daily work? (   )

1. No effect 2. Little effect 3. Some effect 4. Significant effect 5. Extreme effect

B1-6 The following questions are about how you felt about yourself and some other circumstances **in the past week**.

B1-6-1 How much time did you feel calm **in the past week**? (   )

1. All the time 2. Most of the time 3. More of the time 4. Part of the time 5. A small fraction of time 6. No time at all

B1-6-2 How much time did you feel energized **in the past week**? (   )

1. All the time 2. Most of the time 3. More of the time 4. Part of the time 5. A small fraction of time 6. No time at all

B1-6-3 How much time did you feel down and unhappy **in the past week**? (   )

1. All the time 2. Most of the time 3. More of the time 4. Part of the time 5. A small fraction of time 6. No time at all

B1-7 During the past week, how often did your physical health or emotional issue interfere with your social activities? (Such as visiting relatives, friends, etc.)

1. Often 2. Most of the time 3. Sometimes 4. Occasionally 5. Never

B1-8 **In the past 3 months**, have you experienced any of the following situations?

B1-8-1 Eczema (   )

1. Yes 2. No

B1-8-2 Urticaria (   )

1. Yes 2. No

B1-8-3 Allergic rhinitis (   )

1. Yes 2. No

B1-8-4 Food allergies (   )

1. Yes 2. No

B1-8-5 Respiratory infections (   )

1. Yes 2. No

B1-8-6 Catch a cold (   )

1. Yes 2. No (Select this option and answer **B1-9** directly)

B1-8-6a How long was the interval between the last two colds? (   )

1. Less than 1 week 2. 1-2 weeks 3. 3-4 weeks 4. Longer than 1 month

B1-9 Bowel movements **in the past month**.

B1-9-1 How often do you urinate at night? (   )

1. 0 times 2. 1 times 3. 2 times 4.⩾3 times

B1-9-2 Do you have regular bowel movements? (   )

1. Yes 2. No

B1-9-3 How often do you have bowel movements a week? (   )

1.⩽3 times 2. Others

B1-9-4 Do you have trouble defecating? (   )

1. Yes 2. No

B1-9-5 Are you often constipated? (   )

1. Yes 2. No

B1-9-6 Do you feel incomplete emptying when you have a bowel movement? (   )

1. Never 2. Rarely 3. Sometimes 4. Often 5. Always

B1-9-7 Finish a bowel movement need ________(minutes)?

B1-10 The condition of your teeth.

B1-10-1 How many teeth have you lost so far? ____ (If the answer is "0", you can skip **B1-10-3**)

B1-10-2 How many teeth do you currently have available to you? (Including denture) ____

B1-10-3 How many teeth have you lost **in the last 2-3 months**? __________

B1-11 **In the past year**, do you often cough? (   )

1. Yes 2. No

B1-12 When you and other healthy people your age are walking on flat ground, do you get short of breath or slow down because of chest discomfort? (   )

1. Yes 2. No

B1-13 How many falls **in the past year**? (   )

1. No 2.1 to 3 times 3. ⩾4 times

B2 Physical Activity Scale for the Elderly (PASE)

In order to understand your activities in terms of rest time, housework and work **in the past week**, please answer according to the actual situation.

B2-1 Leisure physical activity.

B2-1-1 Over the past 7 days, how often did you participate in sitting activities such as reading, watching TV, or doing handcrafts? (   )

1. None at all (Select this option to answer question **B2-1-2** directly) 2. Rarely (1-2 days) 3. Sometimes (3-4 days) 4. Often (5-7 days)

B2-1-1a What kind of activities did you do? _____

B2-1-1b On average, how many hours per day did you engage in these sitting activities? (   )

1. Less than 1 hour 2.1-2 hours 3.2-4 hours 4. > 4 hours

B2-1-2 Over the past 7 days, how often did you take a walk outside your home or yard for any reason? For example, for fun or exercise, walking to work, walking the dog, walking in a mall, etc? (   )

1. None at all (Select this option to answer question **B2-1-3** directly) 2. Rarely (1-2 days) 3. Sometimes (3-4 days) 4. Often (5-7 days)

B2-1-2a On average, how many hours per day did you spend doing these things? (   )

1. Less than 1 hour 2.1-2 hours 3.2-4 hours 4. > 4 hours

B2-1-2b Over the past 7 days, how much distance did you walk? (   )

1. Less than 1 km 2.1-2 km 3.2-4 km 4. > 4 km

B2-1-3 Over the past 7 days, how many floors did you climb? (One floor has about 10 steps) (
)

1. Less than 1 floor 2. Less than 2 floors 3. Less than 4 floors 4. More than 4 floors

B2-1-4 Over the past 7 days, how often did you engage in light sport or recreational activities such as bowling, golf with a cart, shuffleboard, fishing from a boat or pier or other similar activities? (   )

1. None at all (Select this option to answer question **B2-1-5** directly) 2. Rarely (1-2 days) 3. Sometimes (3-4 days) 4. Often (5-7 days)

B2-1-4a What kind of activities did you do? _____

B2-1-4b On average, how many hours per day did you engage in these light sport or recreational activities? (   )

1. Less than 1 hour 2.1-2 hours 3.2-4 hours 4. > 4 hours

B2-1-5 Over the past 7 days, how often did you engage in moderate sport or recreational activities such as doubles tennis, ballroom dancing, hunting, ice skating, golf without a cart, softball or other similar activities? ( )

1. None at all (Select this option to answer question **B2-1-6** directly) 2. Rarely (1-2 days) 3. Sometimes (3-4 days) 4. Often (5-7 days)

B2-1-5a What kind of activities did you do? _____

B2-1-5b On average, how many hours per day did you engage in these moderate sport or recreational activities? ( )

1. Less than 1 hour 2. 1-2 hours 3. 2-4 hours 4. > 4 hours

B2-1-6 Over the past 7 days, how often did you engage in strenuous sport or recreational activities such as jogging, swimming, cycling, singles tennis, aerobic dance, or other similar activities? ( )

1. None at all (Select this option to answer question **B2-1-7** directly) 2. Rarely (1-2 days) 3. Sometimes (3-4 days) 4. Often (5-7 days)

B2-1-6a What kind of activities did you do? _____

B2-1-6b On average, how many hours per day did you engage in these strenuous activities?

1. Less than 1 hour 2.1-2 hours 3.2-4 hours 4. > 4 hours

B2-1-7 Over the past 7 days, how often did you do any exercises specifically to increase muscle strength or endurance, such as lifting weights or pushups, etc.? ( )

1. None at all (Select this option to answer question **B2-2** directly) 2. Rarely (1-2 days) 3. Sometimes (3-4 days) 4. Often (5-7 days)

B2-1-7a What kind of activities did you do? _____

B2-1-7b On average, how many hours per day did you engage in exercises to increase muscle strength or endurance, such as lifting weights, pushups, or physical therapy with weights, etc.?

1. Less than 1 hour 2. 1-2 hours 3. 2-4 hours 4. > 4 hours

B2-2 Housework

B2-2-1 During the past 7 days, have you done any light housework, such as dusting, washing or drying dishes, or ironing? ( )

1. Yes 2. No

B2-2-2 During the past 7 days, have you done any heavy housework or chores such as vacuuming, scrubbing floors, washing windows, or carrying wood? (   )

1. Yes 2. No

B2-2-3 During the past 7 days, did you engage in the following activities?

B2-2-3a Home repairs like painting, wallpapering, electrical work, etc. (   )

1. Yes 2. No

B2-2-3b Lawn work or yard care, including snow or leaf removal, chopping wood, etc. (   )

1. Yes 2. No

B2-2-3c Outdoor gardening. (   )

1. Yes 2. No

B2-2-3d Caring for another person such as a child, dependent spouse, or another adult. (   )

1. Yes 2. No

B2-3 Work

B2-3-1 During the past 7 days, did you work for pay or as a volunteer? (   )

1. Yes (Select this option to finish the answer) 2. No

B2-3-1a How many hours per week did you work for pay and/or as a volunteer? ____

B2-3-1b Which of the following categories best describes the amount of physical activity required on your job and/or volunteer work? (   )

<u>Category 1</u> "Mainly sitting with slight arm movements" includes examples such as: office worker, watchmaker, seated assembly line worker, bus driver, etc.

<u>Category 2</u> "Sitting or standing with some walking" includes examples such as: cashier, general office worker, light tool and machinery worker.

<u>Category 3</u> "Walking, with some handling of materials generally weighing less than 50 pounds" includes examples such as: mailman, waiter/waitress, construction worker, heavy tool and machinery worker.

<u>Category 4</u> "Walking, with some handling of materials generally weighing considerable heavy" includes examples such as, stonemason, farm or general laborer.

B3 Fatigue Scale-14

B3-1 Do you have problems with tiredness? (   )

1. Yes 2. No

B3-2 Do you need to rest more? (   )

1. Yes 2. No

B3-3 Do you feel sleepy or drowsy? (   )

1. Yes 2. No

B3-4 Do you have problems starting things? (   )

1. Yes 2. No

B3-5 Do you start things without difficulty but get weak as you go on? (   )

1. Yes 2. No

B3-6 Are you lacking in energy? (   )

1. Yes 2. No

B3-7 Do you have less strength in your muscles? (   )

1. Yes 2. No

B3-8 Do you feel weak? Mental symptoms? (   )

1. Yes 2. No

B3-9 Do you have difficulty concentrating? (   )

1. Yes 2. No

B3-10 Do you have problems thinking clearly? (   )

1. Yes 2. No

B3-11 D you make slips of the tongue when speaking? (   )

1. Yes 2. No

B3-12 Do you find it more difficult to find the correct word? (   )

1. Yes 2. No

B3-13 How is your memory? (   )

1. Yes 2. No

B3-14 Have you lost interest in the things you used to do? (   )

1. Yes 2. No

C: Survey on sleep, emotions, and cognitive status

C1 Pittsburgh Sleep Quality Index (PSQI)

The following questions relate to your usual sleep habits during the past month only. Your answers should indicate the most accurate reply for the majority of days and nights **in the past month**. Please answer all questions.

C1-1 During the past month, when have you usually gone to bed at night? USUAL BED TIME__

C1-2 During the past month, how long (in minutes) has it usually take you to fall asleep each night? NUMBER OF MINUTES______

C1-3 During the past month, when have you usually gotten up in the morning? USUAL GETTING UP TIME______

C1-4 During the past month, how many hours of actual sleep did you get at night? (This may be different than the number of hours you spend in bed.) HOURS OF SLEEP PER NIGHT ______

C1-5 During the past month, how often have you had trouble sleeping because you...

C1-5-1 Cannot get to sleep within 30 minutes ( )

1. Not during the past month 2. less than once a week 3. once or twice a week 4. three or more times a week

C1-5-2 Wake up in the middle of the night or early morning ( )

1. Not during the past month 2. less than once a week 3. once or twice a week 4. three or more times a week

C1-5-3 Have to get up to use the bathroom ( )

1. Not during the past month 2. less than once a week 3. once or twice a week 4. three or more times a week

C1-5-4 Cannot breathe comfortably ( )

1. Not during the past month 2. less than once a week 3. once or twice a week 4. three or more times a week

C1-5-5 Cough or snore loudly ( )

1. Not during the past month 2. less than once a week 3. once or twice a week 4. three or more times a week

C1-5-6 Feel too cold ( )

1. Not during the past month 2. less than once a week 3. once or twice a week 4. three or more times a week

C1-5-7 Feel too hot ( )

1. Not during the past month 2. less than once a week 3. once or twice a week 4. three or more times a week

C1-5-8 Had bad dreams ( )

1. Not during the past month 2. less than once a week 3. once or twice a week 4. three or more times a week

C1-5-9 Have pain (   )

1. Not during the past month 2. less than once a week 3. once or twice a week 4. three or more times a week

C1-5-10 Other reason(s), please describe ___________________________

_________. How often during the past month have you had trouble sleeping because of this? (
)

1. Not during the past month 2. less than once a week 3. once or twice a week 4. three or more times a week

C1-6 During the past month, how would you rate your sleep quality overall? (   )

1. Very good 2. Fairly good 3. Fairly bad 4. very bad

C1-7 During the past month, how often have you taken medicine (prescribed or "over the counter") to help you sleep? (   )

1. Not during the past month 2. less than once a week 3. once or twice a week 4. three or more times a week

C1-8 During the past month, how often have you had trouble staying awake while driving, eating meals, or engaging in social activity? (   )

1. Not during the past month 2. less than once a week 3. once or twice a week 4. three or more times a week

C1-9 During the past month, how much of a problem has it been for you to keep up enough enthusiasm to get things done?

1. No problem at all 2. Only a very slight problem 3. Somewhat of a problem 4. A very big problem

C2 The 10-item Kessler psychological distress scale (K10)

<u>In the past 7 days</u> how often ...

C2-1 Did you feel tired out for no good reason. (   )

1. Less than once a week 2. 1-2 times a week 3. 3-4 times a week 4. 5-6 times a week

C2-2 Did you feel nervous. (   )

1. Less than once a week 2. 1-2 times a week 3. 3-4 times a week 4. 5-6 times a week

C2-3 Did you feel so nervous that nothing could calm you down. (   )

1. Less than once a week 2. 1-2 times a week 3. 3-4 times a week 4. 5-6 times a week

C2-4 Did you feel hopeless. (   )

1. Less than once a week 2. 1-2 times a week 3. 3-4 times a week 4. 5-6 times a week

C2-5 Did you feel restless or fidgety. (   )

1. Less than once a week 2. 1-2 times a week 3. 3-4 times a week 4. 5-6 times a week

C2-6 Did you feel so restless that you could not sit still. (   )

1. Less than once a week 2. 1-2 times a week 3. 3-4 times a week 4. 5-6 times a week

C2-7 Did you feel depressed. (   )

1. Less than once a week 2. 1-2 times a week 3. 3-4 times a week 4. 5-6 times a week

C2-8 Did you feel that everything was an effort. (   )

1. Less than once a week 2. 1-2 times a week 3. 3-4 times a week 4. 5-6 times a week

C2-9 Did you feel so sad that nothing could cheer you up. (   )

1. Less than once a week 2. 1-2 times a week 3. 3-4 times a week 4. 5-6 times a week

C2-10 Did you feel worthless. (   )

1. Less than once a week 2. 1-2 times a week 3. 3-4 times a week 4. 5-6 times a week

C3 Montreal Cognitive Assessment (MoCA)

C3-1 Visuospatial /Executive

C3-1a

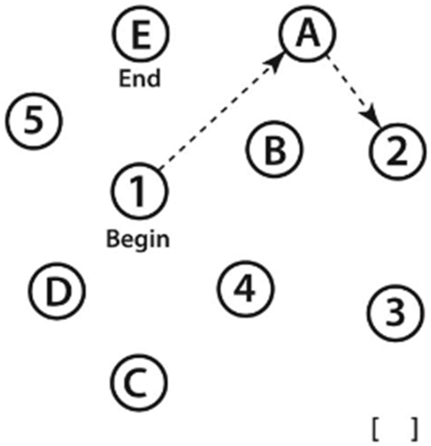

C3-1b Copy Cube

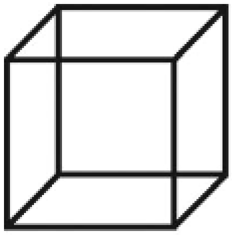

C3-1c Draw CLOCK (Ten past eleven)

C3-2 Naming

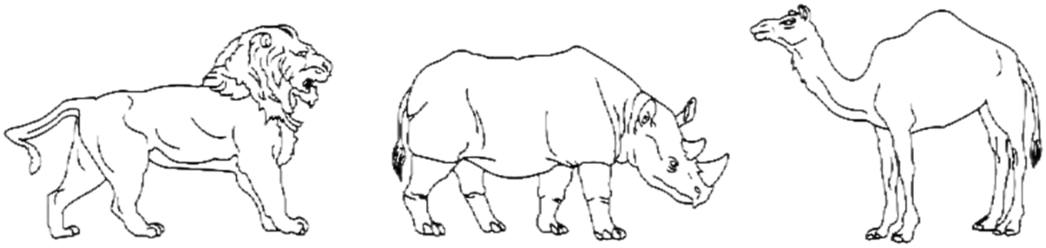

[ ] [ ] [ ]

C3-3 Memory

Read list of words, subject must repeat them. Do 2 trials, even if 1st trial is successful. Do a recall after 5 minutes.

	FACE	VELVET	CHURCH	DAISY	RED
1st trial					
2nd trial					

C3-4 Attention

C3-4a Read list of digits (1 digit/sec)

Subject has to repeat them in the forward order. [ ] 2 1 8 5 4

Subject has to repeat them in the backward order. [ ] 7 4 2

C3-4b Read list of letters. The subject must tap with his hand at each letter A. [ ] F B A C M N A A J K L B A F A K D E A A A J A M O F A A B

C3-4c Serial 7 subtraction starting at 100. [ ] 93 [ ] 86 [ ] 79 [ ] 72 [ ] 65

C3-5 Language

C3-5a Repeat: I only know that John is the one to help today. [ ]

The cat always hid under the couch when dogs were in the room. [ ]

C3-5b Fluency: Name maximum number of words in one minute that begin with the letter F [ ]

C3-6 Abstraction

Similarity between e.g. banana - orange = fruit [ ]train - bicycle [ ]watch - ruler

C3-7 Delayed Recall

Has to recall words WITH NO CUE	FACE [ ]	VELVET [ ]	CHURCH [ ]	DAISY [ ]	RED [ ]
Category cue					
Multiple choice cue					

C3-8 Orientation

[ ]Date [ ]Month [ ]Yea [ ]Day [ ]Place [ ]City

D: Dietary questionnaire

D1 Nutrition literacy questionnaire for the elderly

D1-1 Do you agree with the following statements?

D1-1-1 Reasonable nutrition is not useful for delaying aging and ensuring the health of the elderly. ( )

1. Strongly agree 2. Agree 3. Generally 4. disagree 5. Strongly disagree

D1-1-2 The elderly have reduced digestive capacity and should not consume too much food. ( )

1. Strongly agree 2. Agree 3. Generally 4. disagree 5. Strongly disagree

D1-1-3 The elderly with reduced appetite can make their meals heavier. ( )

1. Strongly agree 2. Agree 3. Generally 4. disagree 5. Strongly disagree

D1-1-4 The elderly is prone to muscle loss, causing falls, disability, leading to a decline in quality of life, should eat more high-quality protein, such as fish, soy. ( )

1. Strongly agree 2. Agree 3. Generally 4. disagree 5. Strongly disagree

D1-1-5 Only children at the stage of growth and development need calcium supplements, and the elderly do not need calcium supplements because they are no longer growing. ( )

1. Strongly agree 2. Agree 3. Generally 4. disagree 5. Strongly disagree

D1-1-6 The elderly should take the initiative to participate in cooking activities such as buying vegetables, washing vegetables, choosing vegetables, and cooking. ( )

1. Strongly agree 2. Agree 3. Generally 4. disagree 5. Strongly disagree

D1-1-7 Eating with family and friends can boost happiness and stimulate appetite. (   )

1. Strongly agree 2. Agree 3. Generally 4. disagree 5. Strongly disagree

D1-1-8 An old man of 100 pounds lost 5 pounds in 30 days, which is a normal weight loss without special attention. (   )

1. Strongly agree 2. Agree 3. Generally 4. disagree 5. Strongly disagree

D1-2 Please classify the following food items into their respective food categories. For example, oranges belong to the category of fruits and vegetables.

D1-2-1 The category of grains and potatoes. (   )

1. Canola oil 2. Citrus 3. Beef 4. Almond 5. Buckwheat

D1-2-2 The category of livestock, poultry, fish and eggs. (   )

1. Canola oil 2. Citrus 3. Beef 4. Almond 5. Buckwheat

D1-2-3 The category of milk, bean and nuts. (   )

1. Canola oil 2. Citrus 3. Beef 4. Almond 5. Buckwheat

D1-2-4 The category of fats and oils. (   )

1. Canola oil 2. Citrus 3. Beef 4. Almond 5. Buckwheat

D1-3 Please recall the food you ate **in the last week** and check the number of types of food you ate **per day** according to the actual situation.

Tips: For example, Mrs. Li ate steamed bread, rice, kidney beans and sweet potatoes yesterday, then she ate 4 kinds of grains, potatoes and mixed beans food; If she also ate apples, Chinese cabbage and tomatoes, then she ate 3 kinds of vegetables and fruits.

D1-3-1 The average daily intake of varieties of grains, potatoes and mixed bean are $\geqslant 3$. (   )

1. Yes 2. No

D1-3-2 The average daily intake of varieties of vegetables and fruits are $\geqslant 3$. (   )

1. Yes 2. No

D1-3-3 The average daily intake of varieties of livestock, poultry, fish and eggs are $\geqslant 3$. (   )

1. Yes 2. No

D1-3-4 The average daily intake of varieties of Milk ,bean and nuts are $\geqslant 3$. (   )

1. Yes 2. No

D1-4 Please recall your diet **in the last week** and answer the following questions according to the actual situation.

D1-4-1 Breakfast. (   )

1. Everyday 2. 5-6 days/week 3. 3-4 days/week 4. 1-2 days/week 5. 0 days

D1-4-2 Coarse grains (including: potatoes, mixed beans, corns, oats, brown rice, etc.). (   )

1. Everyday 2. 5-6 days/week 3. 3-4 days/week 4. 1-2 days/week 5. 0 days

D1-4-3 Fish/poultry/eggs/lean meat. (   )

1. Everyday 2. 5-6 days/week 3. 3-4 days/week 4. 1-2 days/week 5. 0 days

D1-4-4 Vegetables more than 300g. (   )

1. Everyday 2. 5-6 days/week 3. 3-4 days/week 4. 1-2 days/week 5. 0 days

D1-4-5 Fruits. (   )

1. Everyday 2. 5-6 days/week 3. 3-4 days/week 4. 1-2 days/week 5. 0 days

D1-4-6 Milk, yogurt, milk powder or cheese (excluding milk drinks). (   )

1. Everyday 2. 5-6 days/week 3. 3-4 days/week 4. 1-2 days/week 5. 0 days

D1-5 **In the past week**, how many cups of water did you drink per day? (1 cup = 200 ml) (   )

1. ≥7 cups 2. 5-6 cups 3. 3-4 cups 4. 1-2 cups 5. I don't know.

D1-6 **In the past month**, how many days per week have you spent more than 30 minutes of cumulative outdoor exercise? (   )

1. Everyday 2. 5-6 days/week 3. 3-4 days/week 4. 1-2 days/week 5. 0 days

D1-7 How many grams of salt do you think is generally recommended to prevent hypertension? (   )

1. 3g 2. 6g 3. 9g 4. 12g 5. I don’t know.

D1-8 What would you do if a neighbor recommended a health skill? (   )

1. The neighbor's introduction is definitely no problem, completely accept. 2. Depending on their own experience to judge whether it is reasonable and then selectively accept. 3. After consulting professionals, choose to accept according to the response of professionals. 4. Never believe in these health skills, completely do not accept. 5. Don't know what to do.

D1-9 What do you do when a salesman recommends health care products to you? (   )

1. As long as it is a health care product, it is good to eat it directly. 2. Read the health care product instructions carefully to see if it is suitable for you to eat. 3. I will search for information online and choose whether to eat or not according to online recommendations. 4. Consult a doctor, dietitian or other professional before deciding whether or not to eat. 5. Never take any health care products.

D1-10 What is the approximate weight of a medium egg? (   )

1. About 5g 2. About 50g 3. About 100g 4. About 200g 5. I don't know.

D1-11 We usually use the word "handful" to estimate the amount of stem and leaf vegetables. An elderly person over the age of 60 needs to eat 300-500g of vegetables every day, which is equivalent to several handfuls of leafy vegetables. (   )

1. 0-2 handfuls 2. 3-5 handfuls 3. 6-8 handfuls 4. 9-10 handfuls 5. Be ignorant of

D1-12 The following picture shows the nutrition label of a milk. Please read the nutrition label and answer the questions.

In food nutrition labels, "The Nutrient Reference Value (NRV)%" indicates the percentage of nutrient content per 100 grams/milliliters of the food in relation to the daily recommended intake. So, if we rely solely on drinking this milk to get our calcium intake, how much milk should we drink to meet our daily calcium needs? (An estimate is sufficient.) (   )

1. About 200ml 2. 400ml 3. 600ml 4. 800ml 5. I don't know.

Items	per Servings (100mL)	NRV%
**Energy**	277KJ	3%
**Protein**	3.2g	5%
**Fat**	3.8g	6%
**Carbohydrate**	4.8g	2%
**Sodium**	53mg	3%
**Calcium**	135mg	17%

D1-13 When cooking at home, how do you handle meat and vegetables? (   )

1. Vegetables and raw meat should be handled separately from cooked meat and cold dishes by using different cutting boards. 2. Use the same knife to cut vegetables and cold dishes. 3. After cutting raw meat on a cutting board, it can be washed and then used to cut cooked meat. 4. After cutting vegetables with a knife, it can be washed and then used to cut cooked meat. 5. I don't know.

D2 Food Frequency Questionnaire (FFQ)

Please recall whether you have eaten any of the following foods **in the past 3 months**, and estimate the average amount and frequency of these foods.

Food	Food name	1. Yes	Food frequency (select one item only)	Average food

code		2. No	times per day	times per week	times per month	times per year	intake per time (g or mL)
**D2-1 Staple food and beans**							
**D2-1-1**	Instant noodles						
**D2-1-2**	Mixed beans (mung beans/red beans/pinto beans, etc.) (recorded by fresh weight)						
**D2-1-3**	Soybean (yellow bean/green bean/black bean, etc.) (recorded by dry weight)						
**D2-1-4**	Soybean milk						
**D2-1-5**	Tofu						
**D2-1-6**	Fermented tofu (stinky tofu, firm fermented tofu, etc.).						
**D2-1-7**	Ready-to-eat soy products						
**D2-1-8**	Other soy products (including bean sprouts)						
**D2-1-9**	Yuba (including Yuba, oil Tofu skin, etc.) (dry						

	weight)						
**D2-2 Vegetables, fruits and fungi/algae (recorded by fresh weight)**							
**D2-2-1**	Vegetable beans (beans, peas, lentils, Dutch beans, etc.)						
**D2-2-2**	Pickled vegetables						
**D2-2-3**	Fresh fruits						
**D2-2-4**	Edible fungi other than mushrooms (such as black fungus, white fungus, etc.) (recorded by dry weight)						
**D2-2-5**	Mushrooms (entrainmushroo ms, shiitake mushrooms, oyster mushrooms, straw mushrooms, etc.) (fresh weight)						
**D2-2-6**	Laver (fresh weight)						
**D2-2-7**	Kelp (fresh weight)						
**D3 Dried fruits and seeds (recorded by market weight)**							

**D2-3-1**	Peanut						
**D2-3-2**	Melon seeds						
**D2-3-3**	Other nuts (walnuts, pistachios, hazelnuts, etc.)						
**D2-4 Animal food (recorded by edible weight)**							
**D2-4-1**	Livestock and poultry offal						
**D2-4-2**	Fish (including Marine and freshwater fish)						
**D2-4-3**	Shrimp						
**D2-4-4**	Crab						
**D2-4-5**	Molluscs (jellyfish, shell/snail, squid)						
**D2-5 Liquor**							
**D2-5-1**	Wine						
**D2-5-2**	Huangjiu						
**D2-5-3**	Chinese Baijiu						
**D2-5-4**	Beer						
**D2-5-5**	Rice wine						
**D2-5-6**	medicinal wine						
**D2-6 Soup**							
**D2-6-1**	Mushroom soup						
**D2-6-2**	Tremella soup						
**D2-6-3**	Laver soup						
**D2-6-4**	Kelp soup						
**D2-6-5**	Beef/Mutton/Pig						

	offal soup						
**D2-6-6**	Fish soup						
**D2-6-7**	Shrimp meat soup						
**D2-7 Seasoning**							
**D2-7-1**	Salt						
**D2-7-2**	Monosodium glutamate /Chicken essence						
**D2-7-3**	Soy sauce						
**D2-7-4**	Oyster sauce						
**D2-7-5**	Broad Bean Paste)/Fermented Black Beans						

Note: The foods with high nucleotide content are mushrooms, cordyceps, beans, livestock, poultry, offal, aquatic products (fish, shellfish, etc.), fermented foods, reishi. If subjects eat them, make sure to ask about the frequency and intake amount.
